# Profile of gamma-delta (γδ) T lymphocytes in the peripheral blood of crossbreed dogs during stages of life and implication in aging

**DOI:** 10.1186/s12917-020-02504-2

**Published:** 2020-08-08

**Authors:** Cristina Marchetti, Paolo Borghetti, Antonio Cacchioli, Luca Ferrari, Federico Armando, Attilio Corradi, Anna Maria Cantoni

**Affiliations:** Department of Veterinary Science, University of Parma, Strada del Taglio, 10, 43126 Parma, Italy

**Keywords:** γδ T lymphocytes, Dog, Stage of life, Breed, Aging

## Abstract

**Background:**

Data on gamma-delta (γδ) T lymphocytes in the peripheral blood of dogs are scant, related only to healthy pure breed dogs and limited to a restricted age range. The aim of the study was to investigate the modulation of the γδ T lymphocyte (TCRγδ+) subpopulation in peripheral blood of crossbreed healthy dogs according to five identified stages of life: Puppy, Junior, Adult, Mature, Senior and to determine its implication in aging.

A rigorous method of recruitment was used to minimize the influence of internal or external pressure on the immune response. Twenty-three intact female and twenty-four intact male dogs were enrolled. Blood samples were collected and immunophenotyping of peripheral blood T lymphocytes and γδ T cell subpopulations was performed.

**Results:**

The percentage of γδ T cells in peripheral blood lymphocytes was comparable with the value of 2.5% published by Faldyna and co-workers (2001), despite the percentage reported was investigated in less arranged age range groups and coming from four different dog pure breeds, whereas our data were recorded on wider age range groups and coming from crossbreed dogs. Therefore, the γδ T cell percentage (2.5%) is consistent and points out that such value is breed-independent. Statistical analysis highlighted differences in both percentage and absolute γδ T cells according to the stage of life. γδ T cells decreased significantly in the peripheral blood of elder dogs (Senior group) in comparison with previous stages of life (Puppy, Junior, and Adult groups). Differences in γδ T cells are significant and they are reported, for the first time, related to dog aging.

**Conclusions:**

The study confirms dogs to be among the animals with a low TCRγδ+ cell profile. A decrease of the TCRγδ+ subpopulation percentage was observed in elder dogs. TCRγδ+ cells of group S were different from those of groups P, J, and A. The differences are reported for the first time in dog aging. Identifying the stage of life when the decrease of γδ T lymphocytes starts can be useful for providing a rationale for drafting a wellness plan trial to support thymus immune functions and mitigate its functional exhaustion.

## Background

Scientific papers on the lymphocyte subpopulation profile in clinically healthy dogs according to age and sex have been published in large numbers, especially in the last 20 years [1–6]. Data are also available on the gamma-delta (γδ) T cell subset (γδ T lymphocytes) associated with dog autoimmune diseases [7, 8] or tumors [4, 9–13], but currently, there is a lack of data available on γδ T cells in the peripheral blood of healthy dogs. The only data investigating the peripheral blood of healthy pure breed dogs are referred to less arranged age groups [3].

Canine γδ T cells were also investigated in lymphoid organs in early post-natal life [14] and the small intestine revealed the presence in the epithelium of villi, being more numerous in the tip of the villous in comparison with the base [15]. Scattered γδ cells were also observed in the epithelium of crypts, while not detected either in Peyer’s patches or in isolated mucosal lymphoid follicles [15]. γδ T cells mature mainly in the thymus but may even mature in an extra-thymic microenvironment such as the intestine in the mouse [16].

A study on the distribution of γδ cells in the blood circulation defined two different γδ cell profiles: animals with a low population (less than 10%) and animals with a high population (more than 10%) [17]. Dogs, like rodents and human beings, have a low γδ T cell profile (2.5%) [3]. A higher γδ cell fraction (10–90%) is resident within epithelial surfaces, both in the outer epithelial layer (skin) and in the inner luminal epithelium of the body (e.g. gut, and reproductive tract) [18]. An increase of γδ T cell number was observed in bitch uterus mucosa affected by pyometra [19].

The mammalian immune system undergoes dysregulation with aging, a process also known as immunosenescence, which involves age-related changes within central lymphoid organs, detectable as lymphocyte production, migration and function. Thymopoiesis during aging is reduced with thymus involution recognized in human beings as well as in animals [20]. In the canine species, a reduction of the thymic output is age-related and associated with longevity. In large sized short-lifespan pure breed dogs, the thymus cell output decreases earlier within the lifetime of the animal in comparison to small sized long-lifespan pure breed dogs [21]. The distress induces acute thymus atrophy, while inflammation, autoimmune diseases, psychogenic distress conditions, immune suppression, immune stimulation, immunonutrition as well as the body score condition (BSC) interact with, involve and influence the immune response. Likewise, the prolonged physical exercise and heavy training are associated with the suppression of cell function within the immune system [22]. Chronic distress, psychogenic distress, (e.g. confinement, long-term hospitalization, trauma, pain) [23, 24] or immune suppression therapy (e.g. glucocorticoids) can play an important role in determining the efficacy of the canine immune response [25].

Mammals, including the canine species, suffer from the impact of aging, resulting in a decline of the thymic output and reduction of the immune response; moreover, inflammation during aging can impair the immune responses [26]. Having few data currently available investigating the complexity of biology of γδ T lymphocytes within the canine species, this topic presents an intriguing opportunity to define the γδ T cell profile within the peripheral blood in healthy crossbreed dogs according to the stage of life and implication in aging.

## Results

### Dog recruitment

The study was successful in recruiting 47 (23 intact females and 24 intact males) crossbreed healthy dogs, aged from 4 months to 13 years.

Forty-two dogs were recruited from the VTH and the remaining five were recruited from private veterinary practice VTH partners. These cases were recruited over the course of a 2-year period and singular data of 47 recruited dogs are summarized in Table 1.

**Table 1:** Data of recruited dogs according to age, sex, weight and clinical examination

Dog	Age		Sex	Weight	Clinical examination
	Years	Months	Female (F) / Male (M)	Kg	VTH or VTH partners
1	13	0	F	29.0	Routine check-up; Periodontitis stage 3 *
2	13	2	F	30.0	Routine check-up; Periodontitis stage 2
3	7	2	F	36.0	Routine check-up
4	5	3	F	5.0	Neutering
5	12	6	M	12.0	Orthopedic follow-up; Periodontitis stage 2
6	5	4	F	31.0	Ophthalmic follow-up
7	2	4	M	22.5	Ophthalmic follow-up
8	9	2	F	25.0	Routine check-up; Periodontitis stage 2
9	9	5	F	27.0	Routine check-up; Periodontitis stage 2
10	8	3	M	38.0	Routine check-up; Periodontitis stage 2
11	1	6	M	16.4	Microfilaria and antigen testing
12	6	4	M	18.0	Routine check-up; Periodontitis stage 2
13	0	11	F	18.0	Neutering
14	9	0	M	8.6	Routine check-up; Periodontitis stage 3 *
15	3	8	M	57.0	Orthopedic follow-up
16	5	0	M	43.0	Ophthalmic follow-up
17	5	2	F	44.0	Neutering
18	5	5	M	46.0	Ophthalmic follow-up
19	0	11	F	13.0	Neutering
20	1	6	M	22.0	Ophthalmic follow-up
21	4	6	F	48.5	Orthopedic follow-up
22	1	1	M	36.0	Microfilaria and antigen testing
23	2	11	M	25.0	Microfilaria and antigen testing
24	2	6	F	25.0	Ophthalmic follow-up
25	0	8	F	42.0	Neutering
26	1	4	M	37.6	Ophthalmic follow-up
27	1	0	M	39.5	Ophthalmic follow-up
28	0	8	F	16.5	Neutering
29	12	9	M	35.0	Orthopedic follow-up; Periodontitis stage 3
30	10	1	M	32.0	Routine check-up; Periodontitis stage 3
31	0	9	F	5.7	Neutering
32	0	9	M	8.0	Microfilaria and antigen testing
33	0	9	F	18.5	Neutering
34	2	3	F	31.9	Microfilaria and antigen testing
35	0	7	F	30.0	Neutering
36	0	7	F	17.5	Neutering
37	3	10	F	37.8	Ophthalmic follow-up
38	1	3	M	42,0	Ophthalmic follow-up
39	2	11	M	28,0	Microfilaria and antigen testing
40	0	4	M	17.0	Ophthalmic follow-up
41	0	6	M	31.6	Ophthalmic follow-up
42	2	7	M	18.0	Microfilaria and antigen testing
43	8	0	F	31.0	Routine check-up *
44	2	3	F	39.0	Microfilaria and antigen testing
45	1	6	M	17.0	Microfilaria and antigen testing
46	13	3	F	32.0	Routine check-up; Periodontitis stage 2 *
47	10	5	M	23.0	Orthopedic follow-up; Periodontitis stage 3 *

*VTH* Veterinary Teaching Hospital; * dogs recruited from private veterinary practice VTH partners

**Fig. 1:**
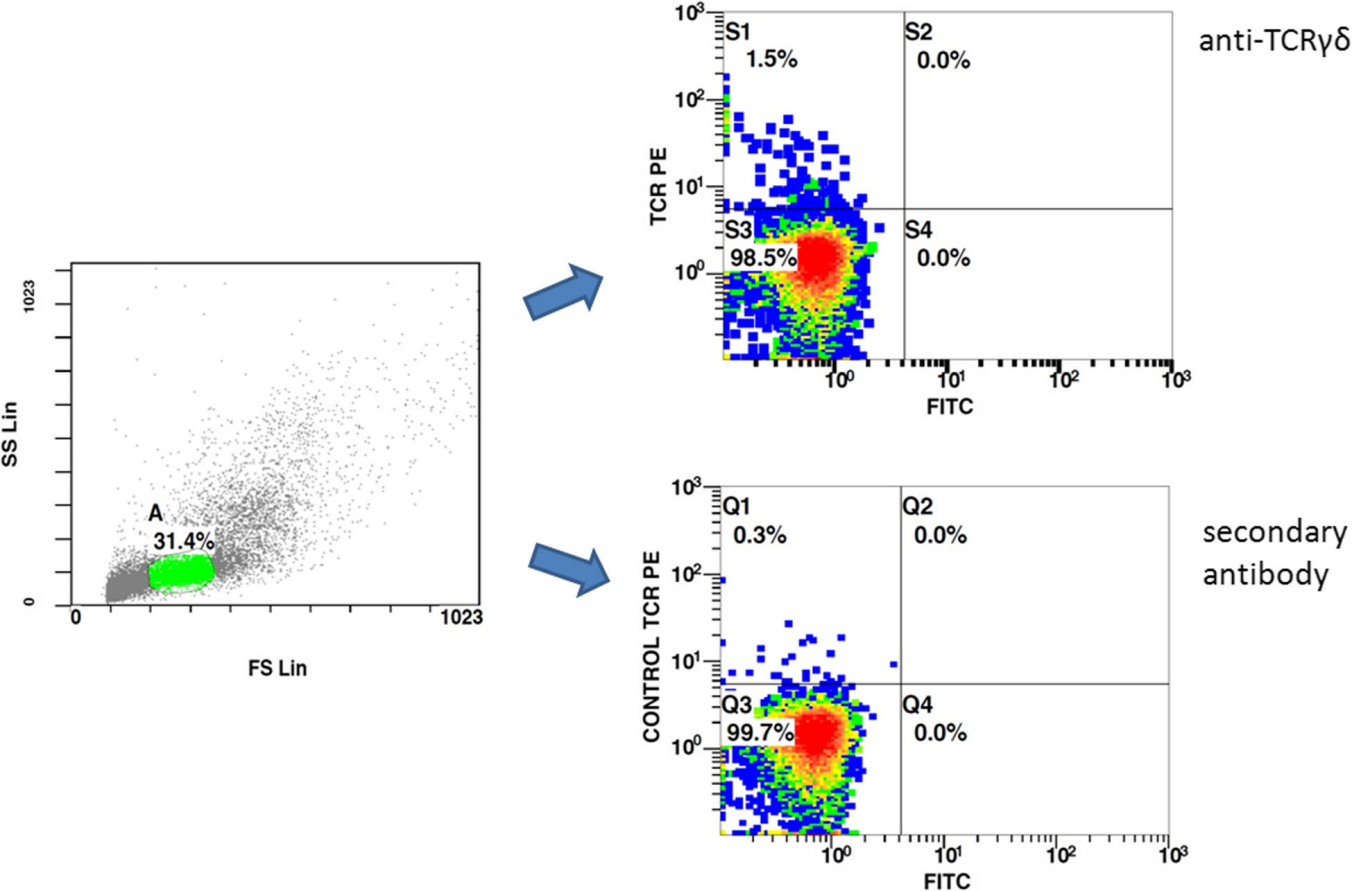
Representative flow cytometry dot plot of canine PBMC, showing lymphocyte gating (A) and density plots of TCRγδ+ cells (left upper quadrant, S1) and unspecific secondary antibody staining (left upper quadrant, Q1)

Data of recruited dogs according to age, sex, weight and clinical examination are summarized in Table 2. All blood samples collected were suitable for flow cytometry analysis (Fig. 1) and cell viability was assessed as > 95% in all samples. Outlier analysis did not provide extreme values deviating from other observations on data.

**Table 2:** Definition of dog groups according to stage of life, also indicating sex and describing weight range and clinical features

	N	Sex	Age	Weight	Clinical Examination
Group P (Puppies)	11 healthy dogs	8 Females 3 males	From 4 to 11 months	From 5.7 to 42.0 Kg	2 males ophthalmological follow-up; 1 male microfilaria antigen-testing; all female referred for neutering
Group J (Junior)	14 healthy dogs	3 females 11 males	From 1 to 2 years	From 16.4 to 42.0 Kg	1 female and 5 males ophthalmological follow-up; 2 females and 6 males microfilaria antigen testing
Group A (Adult)	8 healthy dogs	5 females 3 males	From 3 to 5 years	From 5.0 to 57.0 Kg	2 females referred for neutering; 2 females and 2 males ophthalmological follow-up; 1 female orthopedic follow up; 1 male surgical stabilization after Cranial Cruciate Ligament rupture
Group M (Mature)	7 healthy dogs	4 females 3 males	From 6 to 9 years	From 8.6 to 38.0 Kg	All dogs for routine check-up, 6 showed periodontal disease: 3 females and 2 males stage 2 and 1 male stage 3
Group S (Senior)	7 healthy dogs	3 females 4 males	From 10 to 13 years	From 12.0 to 35.0 Kg	3 females and 1 male routine check-up; 3 male follow-up post traumatic arthropathy. All the dogs affected by periodontal disease: 2 females and 1 male stage2; 1 female and 3 males stage 3

### Quantification of WBC, T lymphocytes and γδ T lymphocytes

**Fig. 2:**
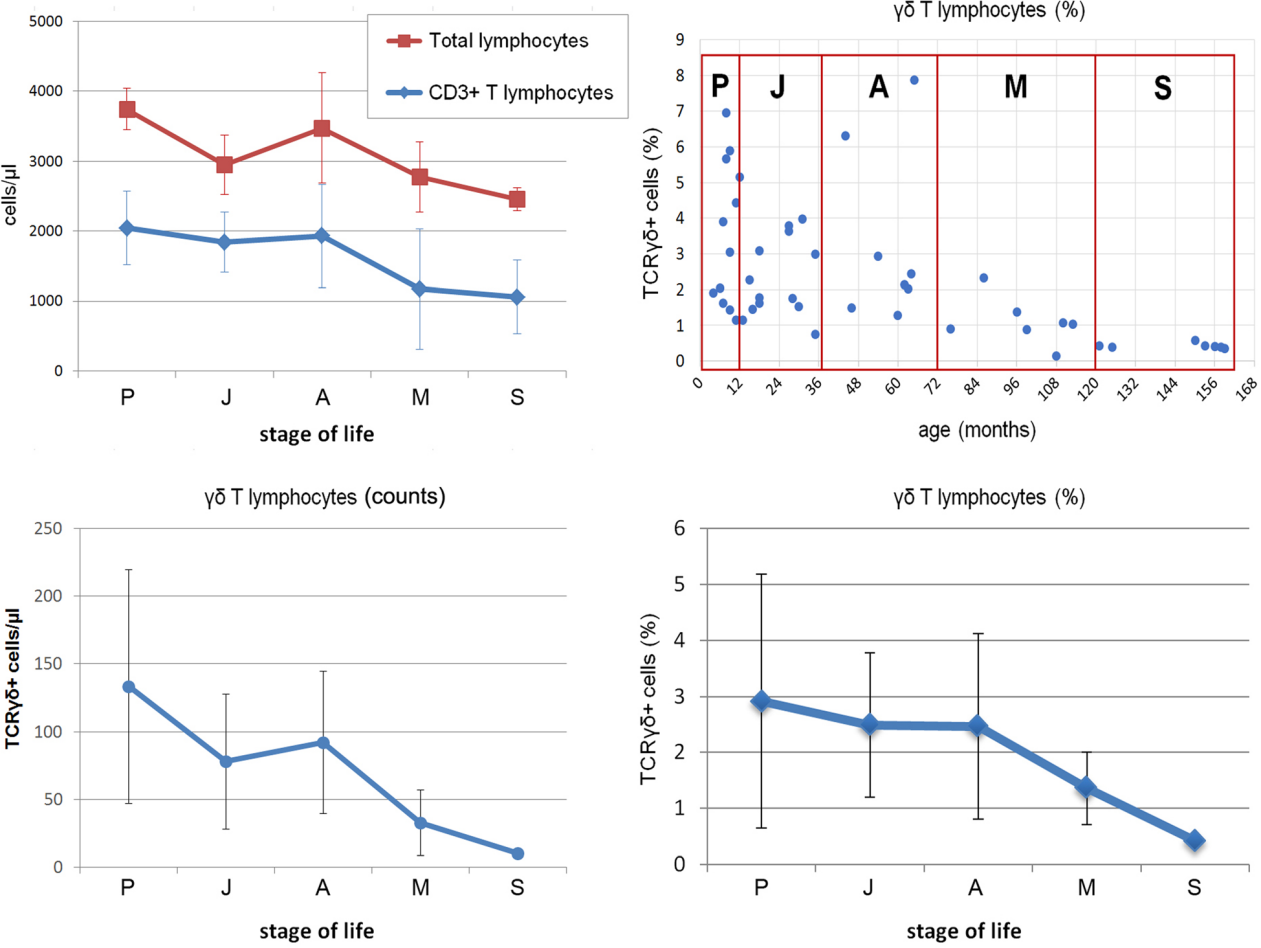
Counts of total lymphocytes and CD3+ T lymphocytes (upper left) and γδ T lymphocytes (lower left) in the peripheral blood of crossbreed dogs in relation to age. Absolute counts are expressed as mean values ± standard deviation (SD). Percentage values of γδ T lymphocytes of each individual dog are shown in relation to the age expressed as months after birth (upper right) as well as to the stage of life (lower right) (P: Puppy, J: Junior, A: Adult, M: Mature, S: Senior)

**Fig. 3:**
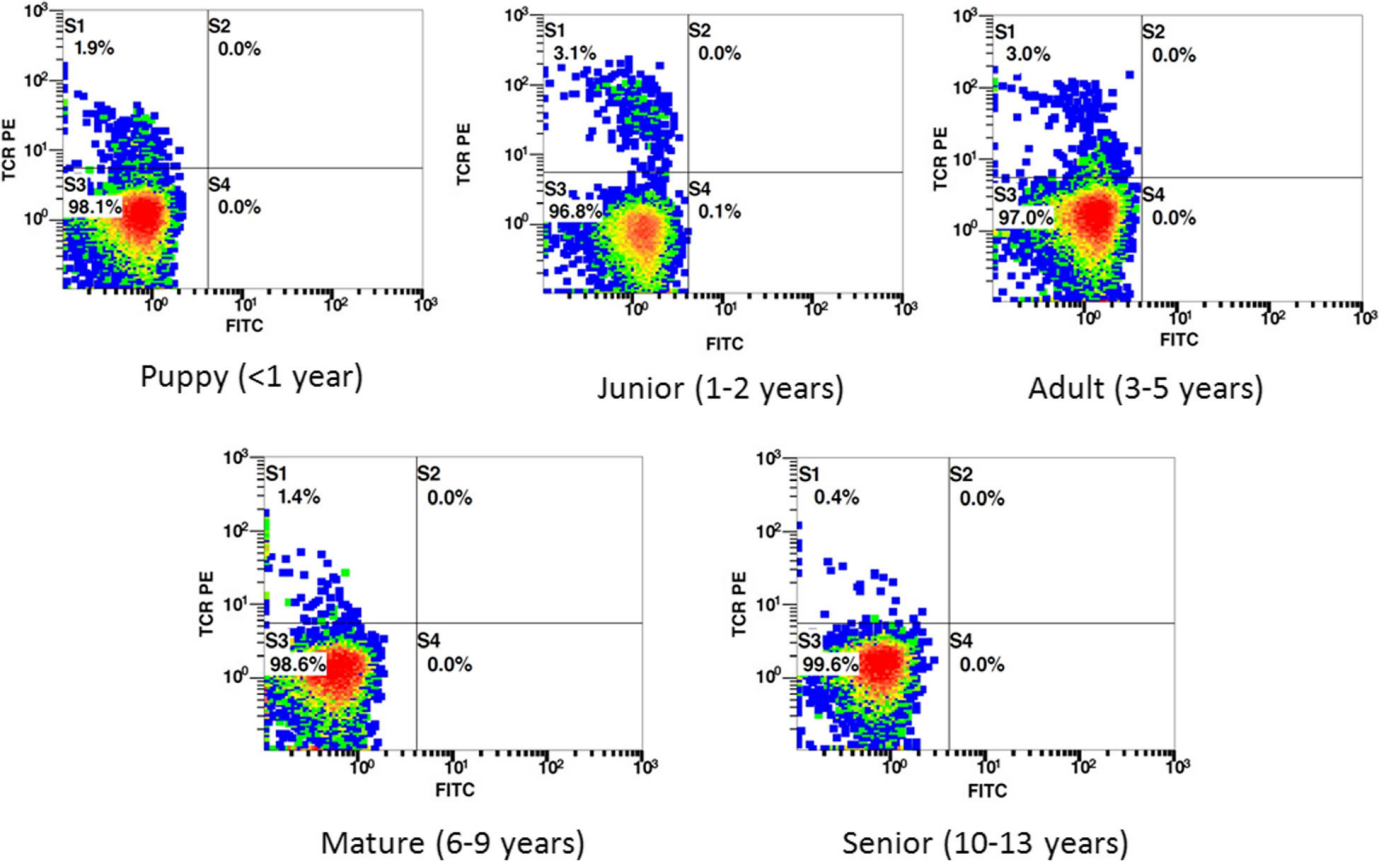
Course of TCRγδ+ cells in peripheral blood of dogs according to stages of life. Representative flow cytometry density plots of canine lymphocyte-gated TCRγδ+ cells (left upper quadrant, S1) are shown according to the five age groups identified in the study (Puppy, Junior, Adult, Mature, Senior). Data are reported as percentage values

Mean values ± SD and range values (minimum and maximum) of white blood cells (WBC), total lymphocytes, and CD3+ T lymphocyte counts and percentages in the peripheral blood of dogs in the different stages of life are reported in Table 3 and Fig. 2. Percentage and absolute mean values ± SD and ranges of TCRγδ+ cells are summarized in Table 3 and shown in Fig. 2 and Fig. 3.

**Table 3:** White blood cells (WBC), total lymphocytes, counts and percentages of CD3+ T lymphocytes and TCRγδ+ T lymphocytes in the peripheral blood of dogs expressed as cell count and percentage mean values ± standard deviation (SD) and percentage range values (minimum and maximum) according to the stage of life

	WBC		Lymphocytes		T lymphocytes (CD3+)				γδ T lymphocytes (TCRγδ+)			
Stage of life	mean ± SD	range	mean ± SD	range	mean ± SD	range	mean ± SD	range	mean ± SD	range	mean ± SD	range
	(cells/μl)		(cells/μl)		(%)		(cells/μl)		(%)		(cells/μl)	
**Puppy (P)**	6509 ± 2502	1600–11,000	3745 ± 294	3400–4400	55.27 ± 15.92	25.60–84.84	2049 ± 523	964–2944	2.92 ± 2.27	1.15–6.94	133 ± 86	40–278
**Junior (J)**	9700 ± 3696	4300–18,700	2950 ± 429	2400–3500	62.99 ± 13.69	22.69–83.64	1839 ± 433	775–2630	2.49 ± 1.29	0.74–3.97	78 ± 50	18–180
**Adult (A)**	10,775 ± 1652	7800–13,600	2968 ± 629	2100–3900	67.35 ± 24.46	14.25–91.60	2037 ± 741	477–2805	2.46 ± 1.66	1.15–6.30	94 ± 53	43–176
**Mature (M)**	8271 ± 996	7000–9500	2771 ± 502	1800–3500	45.56 ± 34.18	8.62–88.70	1180 ± 860	229–2449	1.36 ± 0.65	0.13–2.32	33 ± 24	2–81
**Senior (S)**	7514 ± 358	7000–8000	2475 ± 162	2300–2700	43.05 ± 21.88	6.80–65.70	1056 ± 529	159–1707	0.42 ± 0.08	0.34–0.58	10 ± 3	8–16

All-pairs comparison test for normally distributed data with unequal variances stage of life vs. stages of life, via Tamhane’s T2 test, are summarized in Table 4. Tamhane’s test was also performed for the other immune subpopulations.

**Table 4:** Tamhane’s T2 all-pairs comparison test for normally distributed data with unequal variances stage of life vs. stages of life for percentages of TCRγδ+ cells (significant difference when P ≤ 0.05)

Stage of life	Stages of life	P
Puppy vs.	Junior	1.000
	Adult	0.761
	Mature	0.251
	**Senior**	**0.049**
Junior vs.	Puppy	1.000
	Adult	0.679
	**Mature**	**0.041**
	**Senior**	**0.000**
Adult vs.	Puppy	0.761
	Junior	0.679
	Mature	0.632
	**Senior**	**0.015**
Mature vs.	Puppy	0.251
	**Junior**	**0.040**
	Adult	0.632
	Senior	0.297
Senior vs.	**Puppy**	**0.049**
	**Junior**	**0.000**
	**Adult**	**0.015**
	Mature	0.297

Stage of life of the dogs associated with a *P* value for statistical significance are highlighted in bold characters

The levels of WBC showed a significant increase from Puppy to adulthood and a subsequent decrease in Senior dogs (*p* < 0.05). Both total lymphocytes and CD3+ T lymphocytes showed a significant decrease of cellularity from the young age/adulthood to aged animals (*p* < 0.05) (Fig. 2).

The percentages of TCRγδ+ cells related to the age of dogs independently of the inclusion in one of the five groups identified for classification highlighted a high degree of variation during the first 12 months of life. Variation strongly decreased depending on the growth of the animals. Values resulted the lowest and not dispersed in aged dogs between 120 and 160 months. This course was confirmed analyzing dogs according to the stages of life (Fig. 2). Also the absolute values of γδ T lymphocytes were subjected to a strong decrease in the M and S groups compared to the other age groups.

Comparison between groups of age for TCRγδ+ cell percentages are as follows:

1. Group P vs. Group J, Group A, Group M, Group S

Specifically, a reduction of TCRγδ+ cell percentages, in a slight but constant fashion, from Puppy (group P) was observed by comparison with Junior (group J) and Adult (group A). Figure 2 shows the decline of the percentage of TCRγδ+ cells in the dog peripheral blood during the first three stages of life in which no statistical differences were found (group P vs. group J, *P* = 1.000; group P vs. group A, *P* = 0.761). Figure 2 shows a decreasing percentage of circulating TCRγδ+ cells from Mature (group M, mean = 1.36%) to Senior (group S, mean = 0.42%). The percentage values of TCRγδ+ cells in dogs of group P (mean = 2.92%) compared to those of dogs of group S (mean = 0.42%) were different (group P vs. group S, *P* = 0.049). A reduction of percentages of TCRγδ+ cells in the peripheral blood was detectable during the stages of life from Puppy (mean = 2.92%) to Senior (mean = 0.42%).

2. Group J vs. Group A, Group M, Group S

The percentage values of TCRγδ+ cells of group J (mean = 2.49%) vs. group A (mean = 2.46%) showed a long steady-state period (years) that matches with adulthood of dogs (Fig. 3), and Tamhane’s test was not found to be significant (group J vs. group A - *P* = 0.679). The percentage values of TCRγδ+ cells of group J (mean = 2.49%) compared to those of group M (mean = 1.36%) were identifiably different and Tamhane’s test was significant (group J vs. group M, *P* = 0.041). The phenomenon of decreasing TCRγδ+ cells between group J (mean = 2.49%) and group S (mean = 0.42%) was remarkable and the Tamhane’s test was relevant (group J vs. group S, *P* = 0.000).

3. Group A vs. Group M, Group S

The decline of TCRγδ+ cells between dogs in adult-to-senior stages of life was observed (group A, mean = 2.46%; group S, mean = 0.42%) (Fig. 3). Tamhane’s analysis was significant (group A vs. group S, *P* = 0.015). Group A value of TCRγδ+ cells (mean = 2.46%) was highly different with minus 1.10%, compared to group M (mean = 1.36%). In spite of the change of mean data, no differences were detected by the Tamhane’s test (Group A vs. Group M, *P* = 0.632).

4. Group M vs. Group S

The decline of TCRγδ+ cells in the peripheral blood of dogs of group M (mean = 1.36%) in comparison with Group S (mean = 0.42%) was remarkable (minus 0.94%) but the Tamhane’s test was not significant (group M vs. group S, *P* = 0.297).

5. Group S vs. other Groups (P, J, A, M)

TCRγδ+ cells of dogs of group S were highly different compared to the values of the other groups of dogs (P, J, A, M). Group S was statistically different in comparison with groups P, J, A (group S vs. group P, *P* = 0.049; group S vs. group J, *P* = 0.000; group S vs. group A, P = 0.015), while no statistical difference was detected when group S was tested vs. group M (group S vs. group M, P = 0.297). The graph (Fig. 2) shows the TCRγδ+ cell decline in dog aging. The post-hoc power analysis performed 92.3%.

Taking into account the absolute counts of γδ T lymphocytes in the stages of life, they confirmed what was observed with percentage values. The slight decrease between Puppy and Adult dogs was followed by a significant decrease of cellularity in the group M and particularly in the groups S (*P* < 0.05) (Fig. 2).

## Discussion

The present study was carried out to evaluate γδ T lymphocytes in the peripheral blood of healthy crossbreed dogs according to the stage of life. The recruitment of crossbreed healthy dogs, according to age and categorised according to five groups of stages of life (groups P, J, A, M, S) indicated dynamic changes of the TCRγδ+ subpopulation through the dog lifespan. Major significant findings were observed when comparing dogs in the early stages of life (group P and group J) to those in the later stages of life (group M and group S) so that the declining course of the TCRγδ+ subpopulation was significant. These findings were demonstrated in healthy crossbreed dogs in which both WBC and total lymphocytes as well as T lymphocytes subpopulations were within the normality ranges, and particularly WBC showed a course relative to a normal development of the immune cellularity related to bone marrow-derived cells influenced by aging.

It is to underline that the difference in the numbers of dogs recruited in each group is related to the health condition and the decline of aged class demography. The number of dogs in the Adult, Mature and Senior groups were the most disadvantaged by inclusion and exclusion criteria adopted in the study. The aged dog population (Mature and Senior groups) often did not meet these criteria. Demography of the aged dog population, that spontaneously and naturally decreases, negatively affects group setting up. These conditions can explain first the slowed recruitment speed of dog groups, and secondly, the limited number of dogs, especially, in Mature and Senior groups.

The highest TCRγδ+ cell variation observed in the dogs during the first year of life (Puppy group) can be related to: 1) in general terms, the different extent and course in the development of the immune system after birth after the vanishing of maternal passive immunity; 2) the different development of cells involved in the first lines of defence of the body such as γδ T lymphocytes which exert MHC-unrestricted recognition of antigens; 3) the observation of such aforementioned effects in crossbreed dogs which whole body development and functions can be temporally diverse especially after birth [27].

The thymus reaches its maximum development during puberty and continues to play an immunological role during different stages of life. The decline of TCRγδ+ lymphocytes in the peripheral blood of dogs can be partially associated with the natural aging-dependent thymus involution. A recent study on longevity in the dog performed on thymus lymphocyte output evaluating the recent thymic emigrates (RTE) via signal joint-T cell receptor excision circles (sj-TRECs) concluded that the decline is age-associated when compared to the dog breed [21]. Sj-TRECs participate to VDJ recombination occurring in T cell thymopoiesis after removal of δ gene segments [21]. In order to focus the phenomenon of TCRγδ+ cell drop-off, it is useful to refer to a study performed in aged and centenarian humans [28]. In this paper, a decrease of the TCRγδ+ (δ1 and δ2) cell repertoire related to the increase of TCRγδ+ cell responsiveness to apoptosis stimuli (Fas-α and TNF-α) was recorded in addition to the decrease of the TCRγδ+ cell capability to proliferate. As demonstrated by literature, the complexity of the phenomenon of TCRγδ+ cell decline in the peripheral blood of dogs appears to be complex and influenced by several variables. TCRγδ+ cells in aged and centenarian humans are not involved in the remodelling of the immune system as a result of the T cell decline [28]. Currently, there are no specific investigations on the immune system remodelling in dog aging. Dogs like humans and mice are part of the animal cluster characterized by a low TCRγδ+ cell profile (less than 10%) with genomic similarity for TRG [29]. Therefore, aged dogs may have a remodelling pathway of the immune system in common with aged mice and humans. This pathway could be appropriate for all animal species with a low TCRγδ+ cell profile [30]. Immunonutrients (e.g. arginine, zinc ions) as well as hormones (e.g. melatonin) can play an important role in thymulin biosynthesis and thymus functional restoration in aged mice [31]. Treatment with bovine growth hormone (bGH) in dogs aged 66 months showed to induce histomorphological thymus stimulation but side effects related to anti-bGH immune reaction were observed [32, 33]. Therefore, thymus involution can be considered a consecutive phenomenon related to the plasticity modification of the thymus in the scenario of neuroendocrine-thymus interaction during aging [31, 34]. In elderly humans, chronic low-grade inflammation, also called “inflammaging”, is a real risk factor for the onset of even lethal infections [26]. In aged dogs (Mature and Senior), the weakening of the immune system is the result of aging, leading to side effects such as of the onset of lower-grade infections [6]. Arthritis is one of the most common arthropathies for both species, inducing lameness and consequently reduction of exercise. Less opportunity to exercise combined with a high rich calorie diet increases the risk of obesity in the dog and long-term weight gain induces functional impairment of canine T lymphocytes [35]. Another common effect of aging in the dog is reduced calorie needs related to reduce energy demand (minus 30–40%). Obesity in humans predisposes to the onset of chronic systemic inflammation associated with a decrease of Vγ9/Vδ2 T cells [36]. Therefore, TCRγδ+ cell decrease is inversely related to the body mass index. In obese mice, TCRγδ+ cell reduction was recorded in the skin [36]. Aging, “inflammaging”, and obesity are negative organic conditions that impair the preservation of the immune system homeostasis. Aging itself is not a disease but a status, a daily process of molecular and cellular changes that accumulate over time, resulting in damaging effects on biomolecules (e.g. DNA, RNA, and proteins) and structural components of eukaryotic cells (membrane bound-structures and cytoskeletal matrix). Cumulative cell injury evolves to tissue damage and, at the end, to the loss of organ function.

Inflammation is often an unavoidable clinical condition in aged dogs. In our investigation, many aged dogs (M and S groups) that scored ideal BCS (4–5 score) were affected by periodontium chronic inflammation (periodontal disease, stages 2–3: M group, 6 out of 7; S group, 6 out of 7). Dogs of M and S groups showed a significant peripheral blood TCRγδ+ cell decline, with many of these participants affected by chronic periodontitis diagnosed as stage 2 or 3. Significant differences in peripheral blood TCRγδ+ cells were detected in aged dogs (M and S groups) when compared to the youngest animals (J group). The reduction of blood circulating TCRγδ+ cells could be related to an increase of TCRγδ+ cells resident in the epithelium of inflamed gingival mucosa due to bacterial plaque. Persistence of subgingival bacterial plaque induces firstly chronic inflammation, followed by lesions of mucosa around periodontium (e.g. bleeding). TCRγδ+ lymphocytes are activated in the damaged mucosa (gingivitis) in order to repair epithelial wounds and evoke an inflammatory response with release of cytokines and growth factors. Cytokines released by TCRγδ+ cells are crucial for mucosal integrity and functionality preservation while their high-level release can cause the onset of chronic inflammation [36]. In fact, γδ T lymphocytes are protectors of the epithelium through a dynamic and precise equilibrium of cytokines and growth factor release [36].

## Conclusions

The study confirms the canine species to be among the animals with a low TCRγδ+ cell profile, less than 10%, like rodents and humans. The percentage of TCRγδ+ cells in the peripheral blood of dogs recorded is comparable with the value of 2.5% published by Faldyna and co-workers [3], but the percentage reported by these authors was investigated on less arranged age range groups and coming from four different dog pure breeds (Beagle, German shepherd, Dalmatian, Dachshund), while our data were recorded on wider age range groups and coming from crossbreed dogs. It is important to underline that an identical percentage of TCRγδ+ cells independently recorded by our research team and Faldyna and co-workers under different experimental conditions, makes the percentage value of 2.5% consistent and point out that such value is breed-independent. The most original data observed in the present investigation is related to the significant drastic decrease of the TCRγδ+ subpopulation percentage observed in elder dogs (S group). TCRγδ+ cells of dogs of group S were statistically different in comparison with those of dogs of groups P, J, and A. The differences are significant and reported, for the first time, in dog aging.

Moreover, the common dog and human low TCRγδ+ cell profile as well as life-longevity and genomic similarity for the T cell receptor gamma locus (TRG) can be intriguing for providing a rationale for drafting a wellness plan in the dog, as “non-invasive animal model”, to support thymus immune functions and mitigate its aging-dependent functional exhaustion (e.g. arginine, zinc ion, or melatonin supplementation in the diet associated with periodical not stressful physical activity) also in the perspective of a multidisciplinary approach based on the concept of “One Health - One Medicine”.

## Methods

### Dog recruitment

The recruitment of dogs followed a rigorous method based on inclusion and exclusion criteria for minimizing the internal-bodily and external-bodily conditions that could influence the immune response. In fact, several conditions negatively modulate immunity or induce a stronger immune response: stressors (eustress or distress), genetics (dog breed), drugs, vaccines, autoimmune diseases, immunostimulants, immunomodulants, immunosuppressive drugs or xenobiotics, immunonutrients as well as body weight gain.

A high degree of accuracy of electronic medical records of candidates was a necessary prerequisite for recruiting. Only the dogs of reliable owners were enlisted in the study. The recruitment was achieved through two sample pools: referred dogs at the Veterinary Teaching Hospital (VTH) of the Department of Veterinary Science, University of Parma (Italy) and patients referred to private veterinary practice VTH partners. Recruited dogs were referred for neutering, post-surgical orthopedic follow-up or ophthalmological follow-up.

Dog owners had to have a previously established relationship with the clinical staff, reliable and long-term customers of the VTH or VTH partners. Recruited dogs were then divided into five groups of stage of life (Puppy, Junior, Adult, Mature, Senior).

### Inclusion and exclusion criteria

The Fédération Cynologique Internationale (FCI) recognizes 339 distinct pure breeds [27], therefore it was considered appropriate to obtain the γδ T cell profile from the peripheral blood of crossbreed dogs because they represent the largest number of patients in a veterinary hospital or private clinic. The choice of recruiting only crossbreed dogs was also related to remove the potential influence on γδ T cells in the peripheral blood of pure breeds as an “extra” variable that could affect statistical data (confounding variable).

All blood samples were collected from crossbreed healthy dogs. The inclusion and exclusion criteria for dogs enrolled in the present study are reported in Table 5.

**Table 5:** Information relative to the inclusion and exclusion criteria of the variables considered in the dogs enrolled in the study

	INCLUSION CRITERIA	EXCLUSION CRITERIA
**Informed consent from the owner**	Granted	Not granted
**Breed**	Mixed breed	Pure breed
**Health and Wellness**	Healthy life-style in close contact with the owners	Less than 2 h of exercise each day
		Not required for Puppy
**Diet and Feeding Management**	Commercial standard pet food specific for each stage of life Regular meal time each day	Immunonutrient supplementation (especially zinc ions)
		Irregular feeding routine
		Over- or under-feeding
		Competitive eating
**Nutritional Assessment**^a^	Junior, Adult, Mature, Senior: BCS 4 or 5 out of 9	Puppy in poor body conditions
**Vaccinations**	Regularly performed “vaccination plan” ^b^ (certified only by an electronic medical record)	Any dogs with vaccination status not in line with inclusion criteria Data not certified by an electronic medical record
	Test performed after 3 weeks post-vaccination	
**Therapies, Trauma, Stressors or Imaging Diagnostic Techniques**	After 2 months from:	Data not certified by an electronic medical record Immunomodulant or immunosuppressive therapies Medications, surgeries, trauma and stressors
	medications, surgeries, trauma and stressors	
	X-rays exposure or computed tomography (CT)	
**Sex**	Entire female or male	Female: pregnant, in estrus, any pathological status of the reproductive tract
		Male: cryptorchidism, any pathological status of the reproductive tract
**Parasites**	Endo/ecto-parasites free	Presence of endo/ecto-parasites or therapies in the last 3 weeks
**Periodontal Diseases**^c^	Stages 1, 2, 3	Stage 4 and/or other inflammatory/ulcerative diseases
**Arthritis**	Joints had to be free from any pathological status	Acute and chronic pathological conditions

^a^ A Body Condition Score (BCS) 9-point scale system according to the 2011 World Small Animal Veterinary Association (WSAVA) Nutritional Assessment Guidelines was adopted [37]: 1, 2, 3 = under ideal; 4, 5 = ideal; 6, 7, 8, 9 = over ideal

^b^ According to the 2011 American Animal Hospital Association (AAHA) Canine Vaccination Guidelines [38]

^c^ Periodontal disease was scored according to a periodontal 4-point scale system provided by the Merck Veterinary Manual [39]

### Stages of life and sample size

Recruited dogs were assigned to one of the following five groups based on the stage of life according to age. Puppy: dogs younger than 1 year (group P, *N* = 11); Junior: dogs aged 1–2 years (group J, *N* = 14); Adult: dogs aged 3–5 years (group A, *N* = 8); Mature: dogs aged 6–9 years (group M, *N* = 7);

Senior: dogs aged 10–13 years (group S, N = 7).

No power analysis for recruitment was performed and sample size was based on sample availability. The groups were formed including as many subjects as possible according to the inclusion and exclusion criteria. Specifically, the number of dogs was chosen among the clinical cases managed, in 2 years, at the VTH or referred by private veterinary practice VTH partners. The total number of medical care assisted dogs amounted to 7068 patients (crossbreed and pure breed), of which 4806 (67.99%) were crossbreed. The total of potential selected clinical cases amounted to 52 dogs, but in 5 cases (9.61%) the dog owner did not sign the consent form. Three dogs that could not be recruited were classifiable in the Adult group and the remaining two could have belonged to the Mature group and to the Senior group (one in each group). The recruitment ratio was 0.97%: this percentage was calculated on the total of crossbreed patients.

### Physical examination

During physical examination, clinicians identified the suitable dogs and owners were offered the opportunity to participate in the study. The selected owners were asked for permission for recruitment of the dog by signature of a consent form. Recruited dogs were individually identified by a microchip.

Blood samples were collected from the brachiocephalic vein for routine clinical biochemistry analyses during physical examination. An aliquot was collected in a vacutainer K_3_EDTA tube (Becton Dickinson, USA) for quantification of CD3+ T lymphocytes and γδ T lymphocytes by flow cytometry (FCM).

### Whole blood cell and lymphocyte quantification

White blood cells (WBC) and total lymphocytes were quantified in whole blood samples by using an automated Cell Dyn 3500 plus hematology analyzer (Abbott Diagnostics, Lake Forest, IL, USA) and from blood smears stained with May Grunwald-Giemsa, respectively.

### Isolation of canine peripheral blood mononuclear cells (PBMC)

Blood samples were maintained at room temperature and processed within a maximum of 6 h. Blood was diluted 1:1 with sterile Dulbecco’s PBS + 5% EDTA (Sigma, St. Louis, Missouri, USA). The sample was then stratified on an equal volume of Lympholyte® (Cedarlane®, Burlingtone, NC, USA) and centrifuged at 400 xg for 30 min. PBMC were separated by density gradient, collected and washed twice in 10 ml of sterile PBS + 5% EDTA. The cell pellet was resuspended in PBS + 5% EDTA + 5% heat-inactivated (hi) fetal bovine serum (FBS), quantified and checked for viability by Tryphan Blue exclusion (Sigma-Aldrich, St. Louis, Missouri, USA) by using a TC20™ automated cell counter (BioRad, Hercules, CA, USA) before staining for FCM analysis.

### Flow cytometry analysis

Quantification of T lymphocytes (CD3+) and γδ T lymphocytes within isolated PBMC was carried out by a mouse anti-dog CD3-FITC (clone CA17.2A12, IgG_1_, AbD Serotec, Raleigh, NC, USA) and a mouse anti-dog TCRγδ (clone CA20.8H1, IgG_2a_, Prof. Peter F. Moore, Leukocyte Antigen Biology Laboratory (LABL), Davis, CA, USA [40]; primary antibodies followed by a goat anti-mouse IgG_2a_-PE secondary antibody (cat. M32204; Invitrogen™- Molecular Probes® Carlsbad, CA, USA) according to previously established protocols and optimal signal-to-noise ratio concentration [41, 42]. Each incubation step with the anti-CD3 fluorochrome-labelled antibody and the secondary antibody was done in the dark for 15 min. Unstained cells and cells incubated with the secondary antibody were used as negative controls (non-specific binding). All washing steps were performed with 1 ml of PBS + 1% hi FBS. Analysis was carried out using a Cytomics FC500 MCL flow cytometer and Expo32™ ADC software (Beckman Coulter, Indianapolis, IN, USA) based on lymphocyte gating (forward scatter vs. side scatter) after acquisition of at least 10,000 cell events in the gate. The results were expressed as percentage values of CD3+ cells and TCRγδ+ cells gated on lymphocytes as well as absolute values (cells/μl) based on total lymphocyte counts.

### Statistical analysis

The descriptive analysis was performed for WBC, total lymphocytes, T lymphocytes (CD3+ cells) and γδ T lymphocytes (TCRγδ+ cells). The response variables were total lymphocytes, T lymphocytes and γδ T lymphocytes according to the five stages of life. The significance of stages of life was assessed via the Tamhane’s T2 all-pairs comparison test for normally distributed data with unequal variances. ANOVA one-way and Tamhane’s T2 differences were considered significant if *P* ≤ 0.05. Outlier analysis was performed before the Tamhane’s T2 test. Difference between within-subject and between-subject effects according to stages of life was assessed via post-hoc power with α = 0.05 and significant data were considered if > 80%. Values were presented as mean ± standard deviation (SD) and range, and individual data only for TCRγδ+ cell percentages. All statistical analyses were performed using the SPSS v.25 program for Windows.

### Ethics approval 12345

Blood sample collection was performed after signing the written consent by the owner in compliance with the requirements of the European Directive 2010/63 of the European Parliament and of the Council of September 22, 2010 on the protection of animals used for scientific purposes transposed by the Italian Parliament with “D.lgs Sperimentazione 04/03/2014 n.26”. Chapter 1: General Provisions - Article 1: Subject matter and scope – point 5: this directive shall not apply to the following letter: (f) “practices not likely to cause pain, suffering, distress or lasting harm equivalent to or higher than that caused by the introduction of a needle in accordance with good veterinary practice”. The study was approved by the Ethical Committee of University of Parma, Italy (PROT.N. 20/CE/2019).

## Data Availability

The datasets used and/or analysed during the current study are available from the corresponding author on reasonable request.

## Abbreviations

AAHA: American Animal Hospital Association
ANOVA: Analysis of variance
BCS: Body condition score
bGH: Bovine growth hormone
CT: Computed tomography
δ1 and δ2: Delta 1 and delta 2
Fas-α: First apoptosis signal alpha
FBS: Fetal bovine serum
FCM: Flow cytometry
FCI: Fédération Cynologique Internationale
hi: Heat-inactivated
K_3_EDTA: Tripotassium ethylenediaminetetraacetic acid
Ig: Immunoglobulin
LABL: Leukocyte Antigen Biology Laboratory
PBMC: Peripheral blood mononuclear cells
PBS: Phosphate buffered solution
PE: Phycoerythrin
RTE: Recent thymic emigrates
SD: Standard deviation
sj-TRECs: Signal joint-T cell receptor excision circles
TCRαβ: T cell receptor alpha-beta heterodimer
TCRγδ: T cell receptor gamma-delta heterodimer
TNF-α: Tumor necrosis factor alpha
TRG: T cell receptor gamma locus
VDJ: Variable, diversity and joining genes
Vγ9/Vδ2: Variable gamma 9 / variable delta 2
VTH: Veterinary Teaching Hospital
WSAVA: World Small Animal Veterinary Association
